# Rapid Dissemination of College Food Insecurity Findings in A Multi-Institutional Study Using the eB4CAST Approach

**DOI:** 10.3390/nu12061646

**Published:** 2020-06-02

**Authors:** Melissa D. Olfert, Rebecca L. Hagedorn, Ayron E. Walker, Rachel A. Wattick

**Affiliations:** Division of Animal and Nutritional Sciences, School of Agriculture, Davis College of Agriculture, Natural Resources and Design, West Virginia University, 1194 Evansdale Drive, G25 Agriculture Sciences Building, Morgantown, WV 26506, USA; rlhagedorn@mix.wvu.edu (R.L.H.); aew0036@mix.wvu.edu (A.E.W.); rawattick@mix.wvu.edu (R.A.W.)

**Keywords:** dissemination, visual data, infographic, eB4CAST, college, university, food insecurity, food security, young adults

## Abstract

The sharing of college food insecurity data with higher education administrators and stakeholders is essential to increase awareness of campus-specific food insecurity outcomes. This study utilized the evidence-Based forecast C-capture, A-assemble, S-sustain, T-timelessness (eB4CAST) approach to develop campus-specific food insecurity reports for researchers involved in a multi-institutional food insecurity study. eB4CAST reports were developed for each higher education institution (*n* = 22). The reports were four pages of visual data that included details of the eB4CAST approach and the multi-institutional food insecurity study, campus demographics, an overview of college food insecurity, food insecurity prevalence estimates at all participating institutions, and student use and awareness of campus resources, as well as the campus-specific resources that are available. The interpretation and forecasted use of the reports were evaluated through a 17-item online survey. The survey was completed by 26 content experts and showed a favorable perception of the eB4CAST institutional report. A majority of participants strongly agreed that the eB4CAST food insecurity report was clear to understand (72%), it was easy to read (64%), the statistics were easy to interpret (80%), it shared valuable information (92%), and it was impactful to their work (80%). Further, 84% of participants found the overall information of the report to be relevant and sharable. Participants forecasted disseminating the reports primarily to administration (77%) and with other faculty and staff (85%). These findings highlight the projected usability of the visualized data eB4CAST report across many sectors of college food insecurity research, which may help disseminate rapid findings on this emerging issue and increase awareness.

## 1. Introduction

The landscape of higher education has changed in recent decades, with the cost of attending higher education far exceeding the financial assistance available to students [1,2,3,4,5]. Further, increases in living expenses and related utilities make an already financially restricted situation more dire for students [5]. Consequently, college students are left managing limited budgets and cutting corners to get by, often leading to an increased risk of food insecurity [5].

Food insecurity, or the limited or uncertain access to nutritionally adequate, safe, and acceptable foods, has become a crucial issue for college students in the past decade. A 2020 scoping review found that food insecurity prevalence estimates among college students in the United States (US) range between 10% and 75% and average over 30% [6]. Compared to an 11.1% household food insecurity prevalence nationally [7], the rates among college students are alarming. The impact food insecurity has on college students is also a concern. Studies have shown that college food insecurity is associated with inadequate diet quality [8,9,10,11] along with poor health outcomes, including self-rated health [8,12,13,14,15,16,17,18], mental health, and weight status. Furthermore, food insecurity impacts academic performance, which is counterintuitive to the obtainment of a college degree [8,12,15,16,18,19,20,21]. Thus, it is imperative that higher education institutions understand the magnitude that the impact food insecurity has on college student wellbeing and success.

Despite the influx of research showing the extent and impact of food insecurity on college campuses, there is often a divide between student needs and administrative or stakeholder awareness. Many college campuses have implemented food pantries as the solution to campus food insecurity, yet critics call attention to the inability of food pantries to address the root cause of students’ basic need insecurity [5]. Furthermore, students are often the ones spearheading the campaigns for further assistance for food-insecure students on campus rather than the administration [5]. This calls attention to the potential lack of awareness of the depth of the issue among administration and higher education stakeholders. In fact, administrators are often surprised to learn of the high levels of food insecurity among their students [22]. This points to the need for a greater outreach to administrators and stakeholders to ensure they are aware of the depth of food insecurity on their campus and get involved in decision-making for student food security programming [23]. To do so, there is a need for researchers to have a means of sharing their campus-level data with stakeholders and administrators in a streamlined, understandable manner. However, to date, no research is available on the means of data sharing for food insecurity findings.

The eB4CAST (evidence-Based forecast C-capture, A-assemble, S-sustain, T-timelessness) tool has been developed to facilitate the dissemination of community and campus-based programs to stakeholders [24]. This tool generates a campus or community-specific report that incorporates direct (research results) and indirect (publicly available campus/community level statistics) data to produce electronically-generated infographics that highlight campus or community-specific outcomes and needs [24]. These reports have been used in youth community-based nutrition programming and young adult campus wellness programs, with a positive reception of the usefulness of sharing data with stakeholders [24,25,26]. Thus, eB4CAST could be a means of sharing food insecurity data with higher education administration and campus stakeholders to increase awareness of campus-specific issues. This study aimed to fill that gap and develop an eB4CAST-specific report for researchers who participated in a multi-institutional food insecurity study. The objective of this manuscript was to describe the clarity, relevance, and forecasted use of campus-specific food insecurity eB4CAST reports.

## 2. Materials and Methods

The study design included an online survey used to measure the construction, interpretation, and projected use of eB4CAST food insecurity reports, which are described in the following sections. The survey was sent via email in March 2020 and captured the interpretations of the eB4CAST reports among campus food insecurity researchers in a multi-institutional college food insecurity study. The online survey was conducted using Qualtrics. This study was approved by the university’s Institutional Review Board (IRB) #1611355404.

### 2.1. Participants

An invitation to participate in this study was sent to all researchers who participated in the 2019 multi-institutional food insecurity study lead by (university blinded for review). The 2019 multi-institutional food insecurity study is an expansion of previous collaborative food insecurity research [16] which aimed to assess the prevalence and correlates of basic need insecurities among students in the US. Four institutions had two co-investigators that participated in the multi-institutional study; thus, 26 individuals from 22 higher education institutions were contacted via email to complete an online survey to evaluate the eB4CAST food insecurity reports, which were attached to the invitation to participate email. All the participants were full-time faculty at their respective institution, except for one participant who was a graduate student and adjunct faculty. The participants were not incentivized for their participation.

### 2.2. eB4CAST College Food Insecurity Report

**Figure 1:**
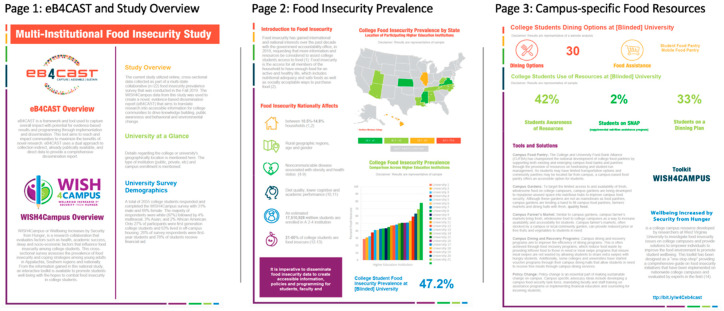
Sample evidence-Based forecast C-capture, A-assemble, S-sustain, T-timelessness (eB4CAST) food insecurity report.

The campus-specific eB4CAST food insecurity reports were designed by the eB4CAST research team with insight from a graphic designer. The eB4CAST team consists of two graduate students, one postdoctoral fellow, and a lead principal investigator with 20+ years of public health and dissemination research. Each report included four pages. Page one provided details on the eB4CAST approach and the multi-institutional food insecurity study as well as campus demographics specific to this study. Page two provided an overview of college food insecurity and showed the food insecurity prevalence estimates at all participating institutions. Page three reported on the student use and awareness of campus food insecurity resources as well as highlighting campus-specific food insecurity resources that are available for students. Page four showcased all the research partner logos and references. The data in each report was customized for each participating university. A sample report is shown in Figure 1.

Figure 1 provides a visual of the eB4CAST food insecurity report for a blinded university. Page 1 provides an overview of eB4CAST and WISH4Campus (Wellbeing Increased by Security from Hunger for Campus; a research initiative to investigate food insecurity and implement best practices on college campuses) as well as an overview of the study and sample. Page 2 provides an introduction to campus food insecurity and associated outcomes along with prevalence outcomes from the multi-institutional study. Page 3 reports on the food resources on campus and the student use of resources. WISH4Campus initiatives are highlighted to provide ideas of new food insecurity programming that could be adopted on a campus. Page 4 is not shown in order to keep the participating universities anonymous.

### 2.3. Survey

The survey used in this study was developed by the authors to specifically evaluate the eB4CAST college food insecurity report and guided by the previous studies using the eB4CAST approach [25]. The survey consisted of 16 items and collected participant demographics, including their academic position, years of involvement in food insecurity activities, and their primary activities related to food insecurity. Further, the survey included a three-point Likert item (strongly disagree, neither agree or disagree, strongly agree) that asked participants’ interpretation of the overall construct of the eB4CAST food insecurity report, which included the clarity, readability, statistical understanding, value, impact, relevance, and shareability of the eB4CAST food insecurity reports, as well as two open-ended questions on where the participants reported the strengths (i.e., most important) and weaknesses (i.e., critiques) of the report. The participants were lastly asked about their project use or dissemination plans to share the report and what sectors (community, administration, faculty and staff, students, etc.) that they planned on sharing the information with and why. A copy of their campus-specific report was emailed along with the survey so that participants could reference the report while completing the survey.

### 2.4. Analysis

The descriptive statistics and frequency calculations based on a three-point Likert item were analyzed using JMP (JMP^®^, Version Pro 13, SAS Institute Inc., Cary, NC, USA, 2015). The open-ended questions were analyzed using a summative content analysis to compile the short feedback comments and quotes [27]. The qualitative data analysis was conducted by two trained researchers, who generated initial codes that informed a coding dictionary and then developed themes to generate summative content to support the quantitative research findings. The independent researchers compared the coding dictionaries to identify discrepancies and collectively agree upon themes.

## 3. Results

### 3.1. Participant Demographics

A total of 26 survey participants were reported from 22 higher education institutions. The respondents reported being teaching faculty (39%), research faculty (19%), both teaching and research faculty (19%), administration (15%), or other (8%), including adjunct positions. The mean length of involvement and experience with food insecurity activities was 9.9 ± 6.7 years. All the participants stated they were involved in college food insecurity research but many were also involved in food insecurity partnership building; program, curriculum, and policy development; information sharing; and awareness initiatives, as shown in Table 1.

### 3.2. Interpretation of eB4CAST Food Insecurity Report

The majority of participants (72%) strongly agreed that the purpose of the eB4CAST food insecurity report was clear to understand, was easy to read (64%), and the statistics were easy to interpret (80%). Thus, survey respondents found there was a clarity of the data presented within the eB4CAST report. Moreover, 92% strongly agreed that the eB4CAST food insecurity report was valuable and impactful to their work (80%). Lastly, 84% of participants found the overall information of the report to be relevant and sharable. Table 2 below provides results from the each three-point Likert item construct studied.

Qualitative data from the open-ended survey questions revealed some strengths and weaknesses of the eB4CAST food insecurity report. Participants found the report to be concise and professional, stating “4 pages is about right in length” and the report was “clean and well-spaced”. The report was stated to be visually appealing, with “colors and layout that are eye-catching”. Participants expressed that the information shared in the report was “important for having conversations with stakeholders”, and it was in a “easy to disseminate format”. The participants highlighted the benefit of showing “how [each] university compares to others in the study”, which was “very helpful and relevant for presenting the findings on [each] campus”.

Nine of the 26 participants (35%) felt the eB4CAST report had all the important information already included, but 65% provided recommendations for improvements. The improvement feedback suggested more specific, individualized and emphasized data for each higher education institution. Furthermore, the participants wanted more comparison data between their university and other campuses in the study, stating that they would “like to see how [their university] compared with other universities in terms of resources and demographics of those who are food insecure”, and that they would like further “comparison to other schools in the state” for universities that had more than one school in a single state. Participants also recommended changing the organization of pages to be more community or administrator-focused and less academic. Specifically, one participant stated “from a community and public standpoint it would make more sense to start with pages 2 and 3 followed by the background with page 1. Another stated “the beginning paragraphs were fairly technical” and may need to be changed to more layman’s terms for use by all stakeholders. They also recommended a “clearer definition in this report of College Student Food Insecurity” for stakeholder understanding. The majority of the recommendation feedback discussed small edits to improve readability and clarity, including the “colors in the graph that show all institutions and percent food insecure are difficult to distinguish” and “using less white space for graphics”. Alphabetizing the institutions was also recommended to improve the cleanliness of the report. Further, the “white text on color background” was noted as hard to read, and the “multiple colors are visually distracting”. A couple of typos were noticed by the participants, and suggestions made to fix these before dissemination use.

### 3.3. Projected Dissemination Plan

The survey examined participants’ projected dissemination or use of the eB4CAST food insecurity report within five key sectors, resulting in the following themes: community, administration, faculty and staff, students, and other institutions. Table 3 reports the percent of participants planning on disseminating within a given sector theme and associated quotes on how they plan to use the eB4CAST report within that sector.

#### 3.3.1. Community Theme

Within the community setting, participants (58%) stated that would share the report with other food insecurity organizations such as food pantries; local non-profits; and other task forces, councils or coalitions. Specifically, participants highlighted that community food security programs are “are well aware of issues of food security, but not in the context of college students”, and these reports can be shared to “raise awareness and to develop strategies for addressing the needs and challenges of students.” Alumni within the community were identified as a potential means to “encourage donations for our new campus food pantry”. Furthermore, local governments were mentioned as a potential dissemination avenue, with the use of this report to “engage with policy makers” who could influence funding for student needs.

#### 3.3.2. Institutional Administration Theme

For institution administration, a large proportion of respondents (77%) indicated that they would share the report with high-level administration, including the University President, the Dean of Students and of different colleges, and student affair representatives. Participants wanted to share with administration to “to inform them of the results from the study that [the university] participated in data collection of” and garner “interest in campus food security initiatives”. Sharing with administration was identified as the main way “to expand on campus services” based on those results and “mobilize resources” to help students in need. Thus, participants stated that this report could help to “make a case for need in programming and accommodations for students” when discussing the issue with administration, and also encourage administration “to send the survey out more widely as a follow-up to get more info, as well.”

#### 3.3.3. Faculty and Staff Theme

The survey participants reported that they would likely engage in peer sharing with faculty and staff (85%) within their department and with partners within other university departments. One participant mentioned that this report should be shared with “all faculty and staff so they have knowledge about these issues that their students are facing”, while another mentioned that it may be best to “share with department heads across our entire campus and allow them to decide whether or not they would like to share with their faculty, staff, and students.” Others identified specific disciplines on campus, including “partners in social work, psychology and public health need to be aware of this growing need so that we can collectively work to find long lasting solutions.” Participants identified that many campuses are developing “groups [of faculty and staff] interested in addressing food insecurity on our campus”, and that this report would be ideal to help them “understand the extent of food insecurity at our university and provide to them along with recommendations for how they can better support students”. Furthermore, participants identified that this report would be important to share with faculty for advising purposes to “be able to advertise available campus resources” and understand the “reality for their students.”

#### 3.3.4. Student Theme

Many respondents (69%) reported their plan to develop a food insecurity curriculum, share with student organizations, and use technological communication for student sharing. Some participants mentioned that would use this report in their current teachings “in order to talk about available resources” in all “relevant classes”. However, some participants mentioned this information should be shared with all students so that they “have knowledge about these issues that their fellow students are facing” and normalize food security programming on campus. Participants identified that “student clubs and leaders need to be aware of this report and findings to help in their collective impact for finding solutions to these problems” and that change can occur if shared with “student government to get more interest in working on initiatives to address food insecurity on campus.”

#### 3.3.5. Other Institutions and Dissemination Uncertainty Themes

A portion of participants (19%) indicated that other institutional sharing presents an opportunity to start new partnerships or strengthen existing ones. Multiple respondents indicated their participation in diverse and multi-sectoral food security partnerships, information sharing networks, task forces, or coalitions and councils as a mechanism to share the eB4CAST food insecurity report. One participant found this to be a good way to engage with “other schools especially those that did not participate in our study” to build relationships for future research. Further, sharing the report with “Cooperative Extension colleagues engaged in food security work” was stated as a means to build a collaborative effort. Others found the report to be a great way to communicate the issues of college food insecurity with national and institutional level organizations that could share the work at a larger scale.

Lastly, a small portion of participants (8%) were uncertain of a dissemination plan for one or all of the analyzed sectors. These participants identified that they thought the report would “help to gain momentum for more institutional support and funding for initiatives to help students”, but that they “have to think about the right audience”. Another participant was not sure if they would “share this particular report”, as they thought their “administration would be mainly interested in only our institution specific info and not some of the other info included on the report.”

## 4. Discussion

This study is the first to look at the rapid dissemination planning of college food insecurity data through the eB4CAST approach. Further, this study gained feedback on a developed visualized data report created as part of a multi-institutional college food insecurity study. With the influx of interest in food insecurity among college students, including attention from the federal government [28], it is essential that data is able to be shared with pertinent stakeholders to raise awareness of the issues college students are facing and justify the need for resources to aid these students [29]. The participants of this study found the eB4CAST report to be clear and easy to understand with valuable and impactful information. The positive perceptions of the eB4CAST reports aligns with previous studies using eB4CAST [24,25,26]. To date, eB4CAST has been used in a childhood obesity intervention in five states [25] and a college health program that occurred at over 50 higher education institutions [26]. Within both of these programs, the eB4CAST approach was favorable among participants, who found the reports to be a feasible means for sharing and disseminating findings back to stakeholders [25,26]. Thus, this study highlights similar findings and strengthens the scalability of eB4CAST into other research areas.

The eB4CAST reports in this study were stated to be sharable, and participants outlined many sectors where they planned to disseminate the reports. Many participants identified multiple sectors they would share the report across, which is ideal as food insecurity programs often require buy-ins from campus and community stakeholders [30,31,32]. The most common sharing sectors were administration, faculty, and staff. This finding is of importance, as these key personnel are often the decision-makers at higher education institutions. However, there is often a disconnect, with many campus stakeholders assuming student needs are being met [33,34]. Furthermore, previous research has shown that students are skeptical of administrators’ commitment to providing basic need resources for students [35], which makes it essential that these players are engaged and understand the impact on their campus to ensure the sustainability of programs on campus. Thus, sharing campus-specific findings among these populations can help to highlight the magnitude of the issue on their campus and influence additional funding from programs [29]. Administrators specifically are recommended to get involved in campus food insecurity initiatives due to their influence over financial decisions [29]; therefore, participants identifying this sector as their planned dissemination route are ideal. For example, McArthur et al. (2018) shared food insecurity research findings with administrators, which led to the development of a campus food pantry and other food insecurity resources [12]. Faculty and staff are also key players who are often directly engaged with the target population. As food-insecure students navigate higher education, engagement with an advisor is a potential avenue for finding support, as often students are not aware resources exist or do not feel comfortable seeking out help on their own [36]. Therefore, faculty and staff are in a unique role to direct students to resources [36], but only if they are aware of the issue and the available resources. By sharing the eB4CAST report with faculty and staff, awareness of the issue on campus can be brought forward, and it also helps faculty and staff to identify resources they can recommend to students in need. Further, the awareness of the issue allows faculty to use real-world data in their course curriculum to increase visibility of the issue to all students [33].

Making students aware of the issue allows them to become a champion of the problem and, as stated previously, people “closest to the problem are closest to the solution” [29]. Students have been at the forefront of creating change on campus, and researchers recommend looking to students as stakeholders [29,32]. Therefore, it is encouraging that participants identified students as a prominent means of dissemination. The participants plan to share the eB4CAST report with students is a vital finding and one that could lead to the development of new student-driven initiatives on campus. Additionally, food-insecure students may be hesitant to make their situation known [33]; thus, sharing the magnitude of the problem with all students may help to make students aware that they are not alone in this problem.

Additionally, engaging with community partners was a projected means of dissemination and aligns with previous suggestions regarding college food insecurity issues. McArthur et al. (2018) stated that community-based approaches including college students could be a “win-win”, in which college students receive a meal and community members get social interaction with college populations [12]. Active participation within different forms of collaboration is an essential component of community-based approaches and facilitates the dissemination of the results to strengthen resources within the community and other institutions [37]. The projected use of eB4CAST aligns with current community-based participatory approaches, with survey participants mentioning sharing the report with different institutions, task forces, networks, councils, or collaborations to strengthen resources and research. Further, utilizing community collaborations provides the inclusion of multiple stakeholders, which can enhance knowledge translation to a diverse audience [37]. Knowledge translation or dissemination has many factors that influence the adoption of evidence-based information, such as clarity, context, perceived values, or meaning [38]. As previously mentioned, eB4CAST provides sharable, impactful, valuable, and clear information which addresses key challenges of dissemination specifically and shows the potential of eB4CAST in successfully translating food insecurity knowledge to diverse institutional and community stakeholders.

Koorts et al. (2020) cited the challenges of translating research into practice, which include resources, organizational support, training or knowledge, time, funding, partnerships, and planning [39]. Effective knowledge translation still remains one of the major obstacles to population health and demonstrates that these challenges still remain [39]. This current study found that 8% of participants did not have a dissemination plan, predominately because the participants did not know an appropriate target audience. This research finding is consistent with dissemination and implementation research within minority or vulnerable populations [40]. Additionally, researchers cite that university students are considered a vulnerable population especially to food insecurity due to the high cost of attending higher education institutions [8]. Yancey et al. (2018) stated that many researchers struggle to identify target populations within low income, rural, or other marginalized groups because of a lack of a plan, support, or resources [40], which is similar to Koorts et al.’s (2020) dissemination challenges [39]. A lack of a dissemination plan or strategy is a passive approach to knowledge translation and creates low intervention adoption rates [41]. When utilized properly, eB4CAST outlines an active dissemination approach to present clear and valuable information to different and diverse sectors that address key vulnerable populations.

Although the feedback was overall positive, participants did have some recommendations for strengthening the eB4CAST reports. The feedback provided key changes, for example adding more emphasis on individual data, which would enhance sharing across sectors as information has to be accessible and applicable for successful dissemination [42]. Further, the specific design and grammatical errors were identified as problems and will be addressed for further dissemination among the partners within this study. There are also limitations to this study. First, to date eB4CAST has been utilized in three research programs targeting community and campus-based populations, but its application in other research avenues needs to be tested for more broad-scale use of the approach. Second, the survey used within this study was developed to specifically assess the eB4CAST report created and was not a validated measure, although it was modified from previously published work. Additionally, only a three-point Likert scale was used with the small sample, so less sensitivity to responses may have been detected. Finally, the participants of the study were experienced and knowledgeable in the issue of food insecurity, and thus the clarity among more layman populations is not known at this time. To improve the study of the eB4CAST approach for college food insecurity, future research should address the perceptions of participants across all sectors and with varying levels of food insecurity experience. Specifically, gaining student input, as they are the at-risk population of the study, would provide valuable insight for higher education stakeholders to understand the barriers students are facing.

## 5. Conclusions

This study is the first to develop a rapid dissemination approach for college food insecurity data and capture planned dissemination from academics to participants in multiple sectors. With the current evidence highlighting the magnitude of the issue on college campuses, it is essential that the findings be shared with key stakeholders. The eB4CAST food insecurity report can be shared to disseminate college food insecurity findings in a visualized story format, with many participants identifying its utility to be shared with administrators, faculty and staff, students, other institutions, and community organizations. This report has the potential to effectively and rapidly increase the awareness of campus-specific issues and be a tool to justify increased funding or the development of new programming to address basic needs for college students. There are limited tools to help translate research into practice, therefore eB4CAST holds the potential to fill this gap. Future research needs to evaluate the effectiveness of eB4CAST and compare the “real-world” utilization to the predicted dissemination plans.

## Figures and Tables

**Table 1:** Respondents’ primary activities related to food insecurity.

Food Insecurity Related Activity	Percent of Respondents	Summarized Examples
Research	100%	-
Building Partnerships and Collaborations	58%	Community academic partnerships; funding and engagement groups
Program Development	35%	Meal kits at campus food pantries; produce prescriptions
Curriculum Development	30%	Courses on food policy and food justice; experiential learning
Policy Development	23%	Drafting local legislation; participating in strategic teams
Information Sharing	38%	Presenting at conferences; publications; social media outlets; meeting with administrators
Strategies for Awareness and Promotion	38%	Hunger free task force; coordinating with student outreach; creating social media to publicize initiatives

**Table 2:** Frequency table (as a percentage) of participants’ interpretations of the eB4CAST food insecurity report.

Construct	Strongly Disagree	Neither Agree or Disagree	Strongly Agree
Clear to understand	4%	23%	73%
Easy to read	8%	27%	65%
Statistics were easy to interpret	-	19%	81%
Valuable	-	8%	92%
Impactful	4%	15%	81%
Relevant Information	-	15%	85%
Shareable Information	4%	11%	85%

**Table 3:** Suggested dissemination plans within sectors.

Sector	Participants (%)	Associated Quotes
Community	58%	“Our local food bank since they helped us to open our campus food pantry.” “Community being that network, which is outside of the university such as SNAP, farmers gardens, grocery stores, etc. These partners need to understand the stark need of our young adults [in college] and I feel they do not know about it.” “I’m working on two taskforces in the community. This type of information would be very beneficial to both groups and will provide an example of how these groups could disseminate information.”
Administration	77%	“Administration needs to be aware of this pressing issue and programs and funds need to be forecasted to help decrease the prevalence.” “I think getting this to as many administrators as possible is important. A couple that come to mind are the Dean of Students and Director of Auxiliary Services…” “This is an important issue and…we have a startling rate of food insecurity [at our university] and I think these administrators would want to know this to help disseminate info about the available services like our pantry and garden.”
Faculty and Staff	85%	“I think [faculty and staff] would like to know that this is a reality for their students and be able to advertise available campus resources.” “…faculty inside the department to facilitate discussions with their students or classes regarding food security.” “I would share with other faculty on campus who are concerned about and/or research food insecurity.”
Students	69%	“I use it to address food insecurity in my sociology of poverty class and other courses in which it could be relevant.” “Students in my department are interested in issues of food access and this report may give them ideas for future research or projects to increase available campus resources.”
Other Institutions	19%	“I will share this with institutional organizations such as the Association of Graduate Programs in Public Health Nutrition and the Maternal and Child Health Nutrition Leadership Grantee directors and faculty.” “I believe there are many other in-state partners that would be interested in this report as well such as the DHHS.” “Similar to the students, this could generate collaboration possibilities with other institutions.”
Dissemination Uncertainty	8%	“I have to think about the right audience.”

## References

[1] Snyder T., de Brey C., Dillow S. (2019). Digest of Education Statistics 2017.

[2] Ma J., Baum S., Pender M., Bell D.W. (2015). Trends in College Pricing. Trends in Higher Education Series.

[3] Mitchell M., Leachman M., Masterson K. (2016). Funding down, Tuition up: State Cuts to Higher Education Threaten Quality and Affordability at Public Colleges.

[4] Mitchell M., Palacios V., Leachman M. (2014). States are Still Funding Higher Education below Pre-Recession Levels.

[5] Freudenberg N., Goldrick-Rab S., Poppendieck J. (2019). College Students and SNAP: The New Face of Food Insecurity in the United States. Am. J. Public Health.

[6] Nikolaus C.J., An R., Ellison B., Nickols-Richardson S.M. (2020). Food Insecurity among College Students in the United States: A Scoping Review. Adv. Nutr..

[7] Coleman-Jensen A., Rabbitt M.P., Gregory C.A., Singh A. (2018). ERR-256. Household Food Security in the United States in 2017.

[8] Farahbakhsh J., Hanbazaza M., Ball G.D., Farmer A.P., Maximova K., Willows N.D. (2017). Food insecure student clients of a university-based food bank have compromised health, dietary intake and academic quality. Nutr. Diet..

[9] Mirabitur E., Peterson K.E., Rathz C., Matlen S., Kasper N. (2016). Predictors of college-student food security and fruit and vegetable intake differ by housing type. J. Am. Coll. Health.

[10] Bruening M., Brennhofer S., van Woerden I., Todd M., Laska M. (2016). Factors Related to the High Rates of Food Insecurity among Diverse, Urban College Freshmen. J. Acad. Nutr. Diet..

[11] Martinez S.M., Grandner M.A., Nazmi A., Canedo E.R., Ritchie L.D. (2019). Pathways from Food Insecurity to Health Outcomes among California University Students. Nutrients.

[12] McArthur L.H., Ball L., Danek A.C., Holbert D. (2018). A High Prevalence of Food Insecurity Among University Students in Appalachia Reflects a Need for Educational Interventions and Policy Advocacy. J. Nutr. Educ. Behav..

[13] Knol L.L., Robb C.A., McKinley E.M., Wood M. (2017). Food Insecurity, Self-rated Health, and Obesity among College Students. Am. J. Health Educ..

[14] Patton-Lopez M.M., Lopez-Cevallos D.F., Cancel-Tirado D.I., Vazquez L. (2014). Prevalence and correlates of food insecurity among students attending a midsize rural university in Oregon. J. Nutr. Educ. Behav..

[15] Payne-Sturges D.C., Tjaden A., Caldeira K.M., Vincent K.B., Arria A.M. (2018). Student hunger on campus: Food insecurity among college students and implications for academic institutions. Am. J. Health Promot..

[16] Hagedorn R.L., McArthur L.H., Hood L.B., Berner M., Anderson Steeves E.T., Connell C.L., Wall-Bassett E., Spence M., Babatunde O.T., Kelly E.B. (2019). Expenditure, Coping, and Academic Behaviors among Food-Insecure College Students at 10 Higher Education Institutes in the Appalachian and Southeastern Regions. Curr. Dev. Nutr..

[17] Hughes R., Serebryanikova I., Donaldson K., Leveritt M. (2011). Student food insecurity: The skeleton in the university closet. Nutr. Diet..

[18] Hagedorn R.L., Olfert M.D. (2018). Food Insecurity and Behavioral Characteristics for Academic Success in Young Adults Attending an Appalachian University. Nutrients.

[19] Raskind I.G., Haardörfer R., Berg C.J. (2019). Food insecurity, psychosocial health and academic performance among college and university students in Georgia, USA. Public Health Nutr..

[20] Martinez S., Brown E., Ritchie L. (2016). What Factors Increase Risk for Food Insecurity Among College Students?. J. Nutr. Educ. Behav..

[21] Silva M.R., Kleinert W.L., Sheppard A.V., Cantrell K.A., Freeman-Coppadge D.J., Tsoy E., Roberts T., Pearrow M. (2015). The Relationship Between Food Security, Housing Stability, and School Performance Among College Students in an Urban University. J. Coll. Stud. Retent. Res. Theory Pract..

[22] Chaparro M.P., Zaghloul S.S., Holck P., Dobbs J. (2009). Food insecurity prevalence among college students at the University of Hawai’i at Mānoa. Public Health Nutr..

[23] Hughes R. (2012). Food insecurity amongst university students. Nutridate.

[24] Olfert M., Hagedorn R., Barr M., Famodu O., Rubino J., White J. (2018). eB4CAST: An Evidence-Based Tool to Promote Dissemination and Implementation in Community-Based, Public Health Research. Int. J. Environ. Res. Public Health.

[25] Olfert M.D., Hagedorn R.L., Barr M.L., Colby S.E., Kattelmann K.K., Franzen-Castle L., White A.A. (2019). Dissemination using infographic reports depicting program impact of a community-based research program: eB4CAST in iCook 4-H. J. Nutr. Educ. Behav..

[26] Olfert M.D., Barr M.L., Hagedorn R.L., Wattick R.A., Zhou W., Horacek T.M., Mathews A.E., Kattelmann K.K., Kidd T., White A.A. (2020). eB4CAST Approach Improves Science Communication With Stakeholders in a College-Based Health Program. Front. Public Health.

[27] Hsieh H.-F., Shannon S.E. (2005). Three approaches to qualitative content analysis. Qual. Health Res..

[28] US Government Accountability Office (2018). Food Insecurity Better Information Could Help Eligible College Students Access Federal Food Assistance Benefits.

[29] Goldrick-Rab S., Richardson J., Schneider J., Hernandez A., Cady C. (2018). Still Hungry and Homeless in College.

[30] Twill S.E., Bergdahl J., Fensler R. (2016). Partnering to build a pantry: A university campus responds to student food insecurity. J. Poverty.

[31] Buch K., Langley S., Johnson T., Coleman N. (2016). A University-Community Partnership to Combat Food Insecurity among College Students. Partnersh. A J. Serv. Learn. Civ. Engagem..

[32] Hagedorn R.L., Pampalone A.L., Hood L.B., Yura C.A., Morrow D.F., Olfert M.D. (2020). Higher education food insecurity toolkit development and feedback. J. Nutr. Educ. Behav..

[33] Stebleton M.J., Lee C.K., Diamond K.K. (2020). Understanding the Food Insecurity Experiences of College Students: A Qualitative Inquiry. Rev. High. Educ..

[34] Dominguez-Whitehead Y. (2015). Students’ food acquisition struggles in the context of South Africa: The fundamentals of student development. J. Coll. Stud. Dev..

[35] Watson T., Malan H., Glik D., Martinez S. (2017). College students identify university support for basic needs and life skills as key ingredient in addressing food insecurity on campus. Calif. Agric..

[36] Diamond K., Stebleton M. (2017). “Do You Understand What It Means to be Hungry?” Food Insecurity on Campus and the Role of Higher Education Professionals. Mentor.

[37] Minkler M., Salvatore A., Chang C. (2012). Participatory approaches for study design and analysis in dissemination and implementation research. Dissemination and Implementation Research in Health: Translating Science to Practice.

[38] Colditz G.A., Emmons K.M. (2012). The promise and challenges of dissemination and implementation research. Dissemination and Implementation Research in Health: Translating Science to Practice.

[39] Koorts H., Naylor P.-J., Laws R., Love P., Maple J.-L., van Nassau F. (2020). What hinders and helps academics to conduct Dissemination and Implementation (D&I) research in the field of nutrition and physical activity? An international perspective. Int. J. Behav. Nutr. Phys. Act..

[40] Yancey A.T., Glenn B.A., Ford C.L., Bell-Lewis L. (2017). Dissemination and implementation research among racial/ethnic minority and other vulnerable populations. Dissemination and Implementation Research in Health: Translating Science to Practice.

[41] Brownson R.C., Colditz G.A., Proctor E.K. (2017). Dissemination and Implementation Research in Health: Translating Science to Practice.

[42] Wallerstein N., Duran B. (2010). Community-based participatory research contributions to intervention research: The intersection of science and practice to improve health equity. Am. J. Public Health.

